# A rapid freezing method to determine tissue layer thickness in drought‐stressed leaves

**DOI:** 10.1111/jmi.13272

**Published:** 2024-01-28

**Authors:** Maryam Alsadat Zekri, Carina Leimhofer, Nicole Drexler, Ingeborg Lang

**Affiliations:** ^1^ Faculty of Life Sciences Department of Functional and Evolutionary Ecology, University of Vienna Vienna Austria; ^2^ Vienna Biocenter Core Facility GmbH Vienna Austria

**Keywords:** *Arabidopsis thaliana*, drought stress, genotypes, mesophyll, plunge freezing, SEM

## Abstract

Plants have been affected by water stress ever since they settled on dry land. In severe and persisting drought, plant leaves are wilting. However, a documentation at the anatomical level of the minute changes that occur before wilting is challenging. On the other hand, understanding the anatomical alteration in plant leaves with respect to water stress provides a stronger basis to study molecular and submolecular processes through which plants enhance drought tolerance. In this work, we applied an affordable method to visualise mesophyll layers of *Arabidopsis thaliana* cell lines without preparation steps that would alter the volume of the cells. We rapidly plunge‐froze the leaves in liquid nitrogen, cut them while in the N_2_ bath, and immediately imaged the mesophyll cross sections in a scanning electron microscope. We applied a reduction of watering from 60 to 40 to 20 mL per day and investigated two time points, 7 and 12 days, respectively. Interestingly, the overall thickness of leaves increased in water stress conditions. Our results showed that the palisade and spongy layers behaved differently under varying watering regimes. Moreover, the results showed that this method can be used to image leaf sections after drought stress without the risk of artefacts or swelling caused by contact to liquids as during chemical fixation.

## 1 INTRODUCTION

As sessile organisms, plants are used to changes in the environment and have adopted several mechanisms, ranging from metabolic processes and signal transduction pathways to morphological responses.^1^ Both, soil properties (pH, salinity, soil porosity) as well as atmospheric conditions such as exposure to sunlight and CO_2_ concentrations greatly influence a plant's productivity.^2^ Here, we focused on water deficiency as an abiotic stress factor.

Plants are capable of handling daily fluctuations in water supply and combat drought conditions for some time. There is a threshold when exceeding water or drought stress can damage plant tissue. If the loss of water exceeds its uptake, plants start to wilt. The ‘permanent wilting point’ is an indicator for the irreversibility of cell damage due to drought stress. In the literature often a value of −1.5 MPa is found, which means a water loss of 30%.^1^ At this stage, the plant's vitality decreases, resulting in higher susceptibility to pathogens, visual morphological changes like reduced growth and yield, and eventually death. Thus, cell turgor and a hydrostatic skeleton is essential for normal plant growth.

The anatomical properties of plants can be modified to facilitate their adaptation to long and/or short‐term environmental water fluctuations.^3^ Changes in stomata density,^4^, ^5^ the thickness of the cuticle,^6^, ^7^ rearrangement of parenchyma cells^8^, ^9^ or trichome density^10^ are some examples of how anatomical properties optimise physiological responses.

Even though plants may change their anatomy to cope with environmental fluctuations, implementation of such changes needs time. Therefore, the numerous observations implying the role of initial reactions and immediate switches in anatomy due to water stress cannot be underestimated. It has been frequently reported that stomata density (SD) and stomata size (SS) have a huge contribution in physiological responses and adaptations of plants.^11^, ^12^, ^13^, ^14^ Due to higher cell surface to volume ratio, smaller stomata respond faster to humidity changes^11^ and stomata density optimises photosynthesis in fluctuating environmental CO_2_ concentration and humidity.^15^ The general idea implies that intercellular air space is proportional to the stomata density and size. It has been documented that a protein called Stomagen is synthesised in the mesophyll and induces stomata density in the epidermis.^16^, ^17^, ^18^ Therefore, environmental stresses such as drought stress may change the anatomy of the epidermis through affecting the mesophyll dynamics.

The thickness of the leaf mesophyll mainly comprises the layers of palisade and spongy parenchyma. The spongy parenchyma is commonly known as a layer with larger intercellular air spaces, which makes this layer more involved in leaf gas exchange and transportation.^19^ The palisade layer is characterised by densely packed and elongated cells with less intercellular space. The palisade cells are more responsible for photosynthesis.^20^ The effect of the whole leaf thickness on carbon assimilation has been show before,^21^ as well as the role of the volume of intercellular air represented by the thickness of the spongy layer,^22^ and the thickness of the palisade layer on photosynthesis.^23^ Still, how the anatomy of the mesophyll may alter during drought stress is not yet clear. One possible reason for this ambiguity is that in most studies, the mesophyll thickness was evaluated with light microscopy techniques.

Light microscopy is usually the method of choice because the cells and tissue should be alive to follow the dynamic processes. However, sample preparation and investigation in water is not feasible for drought‐stressed tissue due to immediate water uptake by wilted cells. X‐ray Microcomputed Tomography (µCT) with high beam intensity, for example, at a synchrotron light source, showed to be very efficient in image acquisition of fresh leaf segments at high resolution within subseconds.^24^ However, this technique requires accessibility to the light source facility and is very expensive. To solve this dilemma, we looked for a cryo‐immobilisation technique to rapidly freeze drought‐stressed leaves and investigate the respective tissue layers in their near‐to‐native state in a scanning electron microscope, without chemical fixation or staining. That way, we succeeded in the determination of water loss at the tissue level in drought‐stressed Arabidopsis leaves. To test our simple preparation method and to investigate morphological changes in leave tissue layers, we froze leaves of four different *A. thaliana* genotypes with varying stomatal traits: clusters of stomata, increased number of stomata, or stomata on both sides of the leave. We aimed to measure the effects of water loss as differences in thickness of (a) the whole leaf, (b) the palisade and (c) the spongy mesophyll. Our main objectives in this work are: (1) to evaluate the accuracy of cryo‐ immobilisation to investigate the minute changes in size of mesophyll layers after different watering regimes and (2) to investigate the effect of drought intensity (moderate and severe) and duration (short and long‐term) on the thickness of the palisade and spongy parenchyma layers. We hypothesised that the different mesophyll layers react differently to water loss. Additionally, we want to test the hypothesis that under moderate stress for a short time, the thickness of the leaf may increase to elevate the photosynthesis rate thereby enabling a plants’ resistance to lack of water.^2^, ^26^

## 2 MATERIAL AND METHODS

### 2.1 Plant material

Four genotypes of *Arabidopsis thaliana* (Col‐8 as wild type, *tmm1*, *lcd1‐1* and UBP15‐overexpression) were ordered from the Nottingham Arabidopsis Stock Centre.^27^ Based on the NASC database, the genotypes were selected based on distinctive anatomical features. *tmm1* (*too many mouths* (tmm)) mutant has clusters of stomata^28^ with significantly higher stomata count than wild type. In *lcd1‐1*, growth is decelerated due to a low cell density (lcd) in the palisade layer.^29^ UBP15 is a transgenic line that grows much bigger than the wild type and its leaves are more curled. Epidermis cells and palisade parenchyma cells are increased per area (The Arabidopsis Information Resource (TAIR)). Col‐8 was selected as the wild‐type or the background line.

Three plants per each genotype were grown in separate pots (diameter = 13 cm) containing 50% indoor plants’ soil (Kranzinger GmbH, Austria), 33% quartz sand, and 17% Perlite® at stable conditions in a growth room (Conviron MTPS 120, Manitoba, Canada) and watered daily with 60 mL water for a cultivation period of 5 weeks. Based on the size of the pots and soil composition, 60 mL was the maximum volume of water that remained in the soil and did not drain from the pots. All the pots were fully saturated with water and left for 1 day before sowing the seeds. The temperature at day was 22.2°C and at night 18°C, relative humidity (RH) was 60% and light intensity 130–150 µmol/m^2^ s. The photoperiod of the plant chamber was set to 16 h day length.

### 2.2 Stress experiments

After 5 weeks, the plants of the control group were continued to be watered daily with 60 mL water as before (Treatment group 1; control). Treatment group 2 received reduced water of 40 mL daily and treatment group 3 only got 20 mL daily. The relative soil humidity was measured with moisture meter (HH2, Delta‐T Devices) after 7 and 12 days. The relative soil humidity after irrigation with 60, 40 and 20 mL, respectively, was ∼75%, 50% and 30%, respectively. According to Rivero et al. (2014), the 75%, 50% and 30%, relative soil humidity are correlated to high, mild, and low humidity, respectively.^30^ All other parameters in the growth chamber were kept constant. After 7 days of this watering regime, one leaf of each genotype from each treatment group was selected for plunge freezing and consecutive scanning electron microscopy (SEM). This time was considered as short‐term stress. After 12 days, which was considered as long‐term stress, leaves from all genotypes and all treatment groups were taken again for plunge freezing and imaging. Selection criteria for the leaves were as follows: similar size, stemming from first or second apical nodus, not touching bare soil or the pot (Figure 2).

### 2.3 Sample preparation for SEM

High resolution and exact measurements of the hydration status in drought‐stressed tissue was essential for this study. We excluded chemical fixation to eliminate the risk of chemically induced morphological changes like swelling or shrinkage.

**FIGURE 1:**
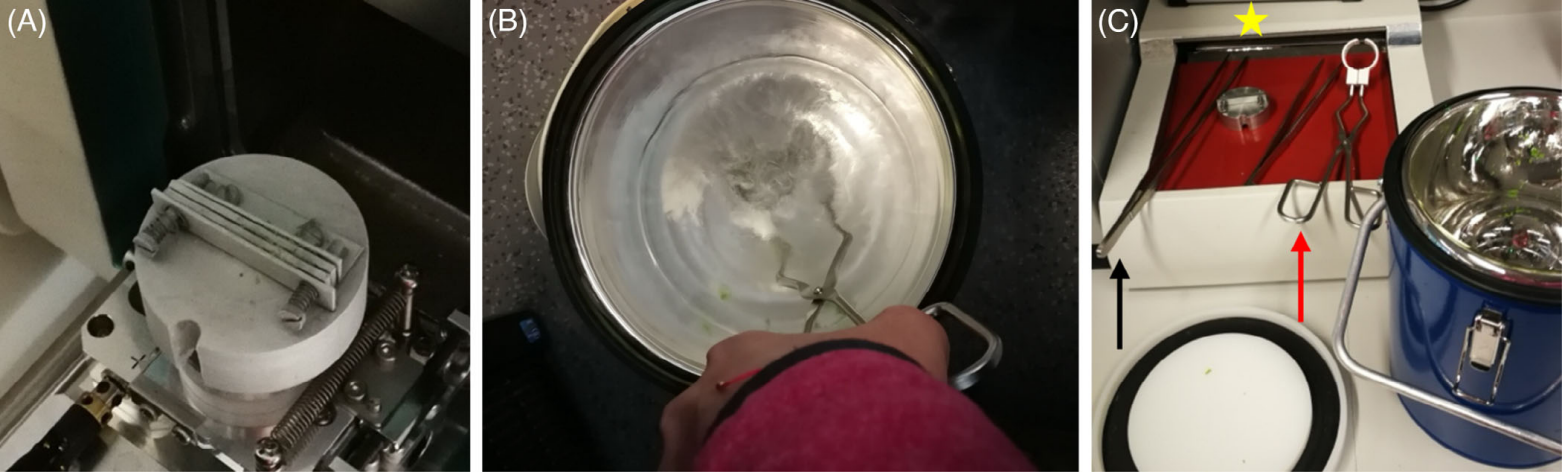
Sample preparation for EM: (A) custom‐designed holder with three plunging spots; (B) the custom‐built holder submerged in liquid nitrogen to freeze the mounted leaves; (C) the forceps used to immerse the leaf holder into liquid nitrogen (red arrow), and to hold the razor blade to cut the leaf while submerged in liquid nitrogen (black arrow), the heater to remove ice from the tools (yellow star).

From each genotype and treatment, two fully developed leaves with no sign of rapture or abnormality were selected and cut from the plant with a razor blade. They were mounted along the leaf distal axis on a custom‐built holder for SEM (Figure 1A) and immediately plunged into liquid nitrogen to preserve leave ultrastructure. Subsequently a razor blade was used to create a sharp lateral‐section of the leaf while still submerged in liquid nitrogen (Figure 1B). Figure 1C depicted all equipment that are required for sample preparation including holder forceps, long forceps, heater and liquid N flask. Frozen samples were immediately transferred to a Hitachi TM‐1000 tabletop scanning EM operated at 15 kV and equipped with a highly sensitive semiconductive BSE detector (https:// www. scribd. com/ document/ 501503982/Manual‐TM1000). The low‐vacuum condition needed for detectors in the TM‐1000 did not require any specimen coating; therefore, no sputter‐coating was applied in the present work. Pictures were taken at a magnification of 600× or 800×, respectively. The middle of the leaf, that is, approximately the same distance from the main leaf vain and the leaf edge, was considered to take three images next to each other of each leaf.

### 2.4 Tissue traits

**FIGURE 2:**
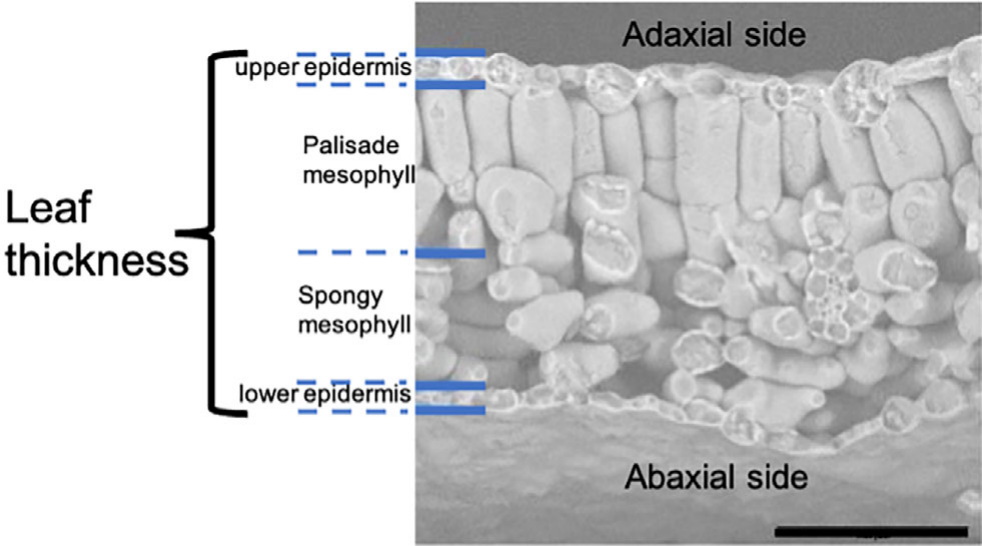
SEM micrograph of a cross section of Col‐8 genotype of A. thaliana. Individual tissue layers are indicated by blue lines; longest palisade cells define the thickness of palisade layer (e.g. cell in the centre of this image). The thickness of palisade layer, spongy layer and total thickness including the epidermis were measured. Bar = 100 µm.

We used two leaves per plant and took three images of each leaf. In each leaf, we took three measurements from each layer and also measured the overall thickness. Thus, in each image, the total thickness of the leaf, the palisade layer and the spongy layer were measured in three spots. The border between the palisade layer and spongy layer was drawn by considering all the palisade cells’ sizes (the shortest and longest palisade cell) in Fiji (Fiji3, 2022). We calculated the mean of the three measurements in each photo: (1) diameter of whole leaf, (2) thickness of palisade layer and (3) thickness of spongy parenchyma layer (Figure 2). For all parameters, we used the program Fiji (Fiji3, 2022).

## 3 RESULTS

**FIGURE 3:**
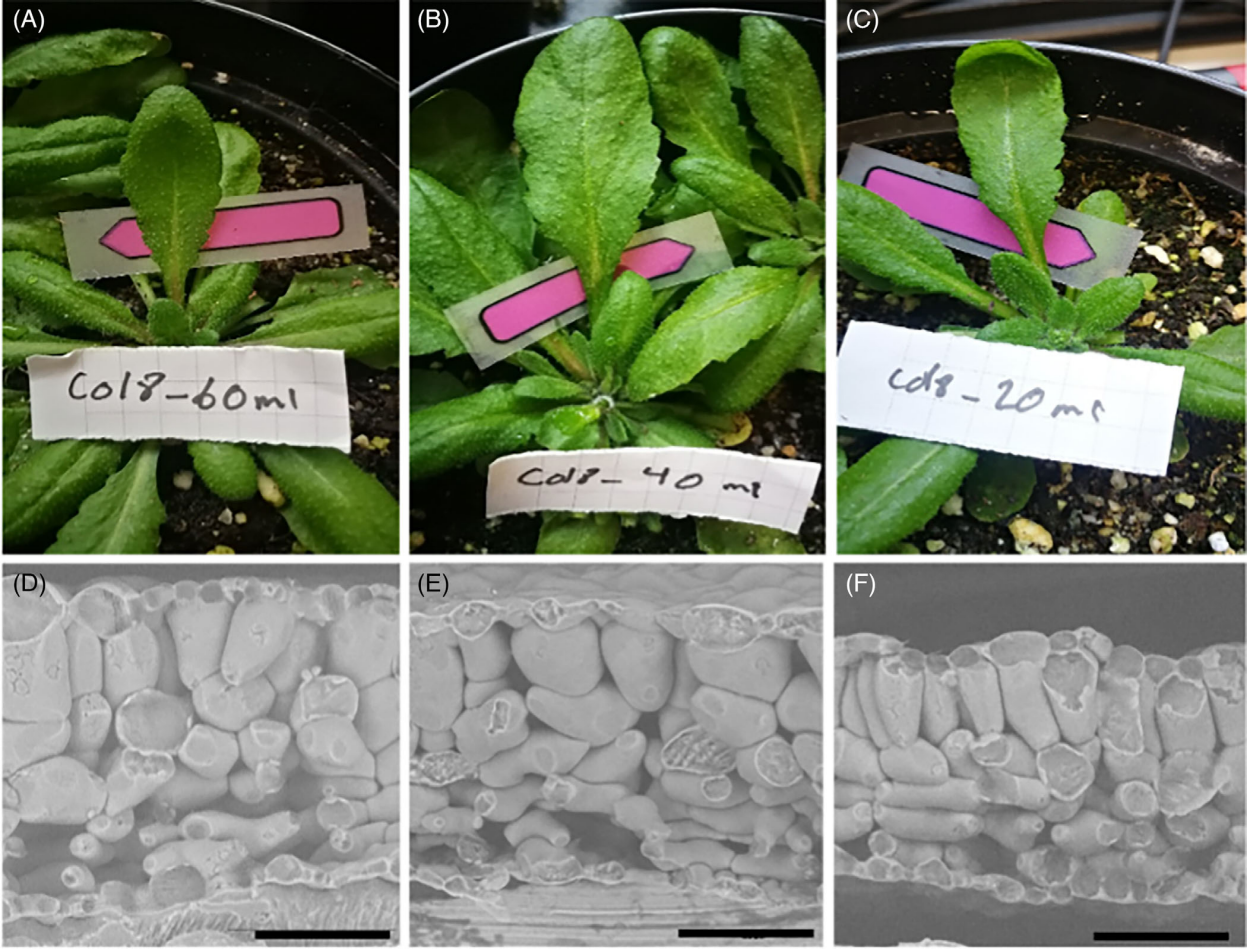
Col‐8 (wild type) after long‐term stress (12 days). A–C shows the Col‐8 plants; selected leaves for freezing are indicated. The original watering regime was 60 mL (A), 40 mL (B) and 20 mL (C). D–F: Cross sections of selected leaves A–C. Note the shrinking of the whole leaf and the increasing cell density at the tissue level in the mesophyll. Bar = 100 µm.

One leaf of all four genotypes of *A. thaliana*, namely, *tmm1*, *lcd1‐1* and UBP15‐overexpression, as well as *Col‐8* (wild type) was carefully cut, placed in the SEM‐sample holder and immediately plunge‐frozen in liquid nitrogen. As an example, Figure 3 shows the leaves of plants of the wild type (Col‐8) during the stress experiment watered with 60 mL (Figure 3A), with 40 mL (Figure 3) and 20 mL water, respectively (Figure 3C). The corresponding cross sections of the selected leaves after plunge freezing are imaged directly in SEM without coating (Figure 3D–F).

### 3.1 Macroscopic changes

Over the course of the experiment, the plants subjected to drought stress showed in poor development and morphological alterations. In Arabidopsis line *tmm1*, we observed an unspecific, yellowish discoloration at the leaf margins in all treatment groups. Plants of line *lcd1‐1* were overall smaller than in the control group and they felt firm and stiff; some leaves showed yellow margins. UBP15 plants were wilting quickly at high water stress (20 mL water).

### 3.2 Short‐term stress (7 days)

**FIGURE 4:**
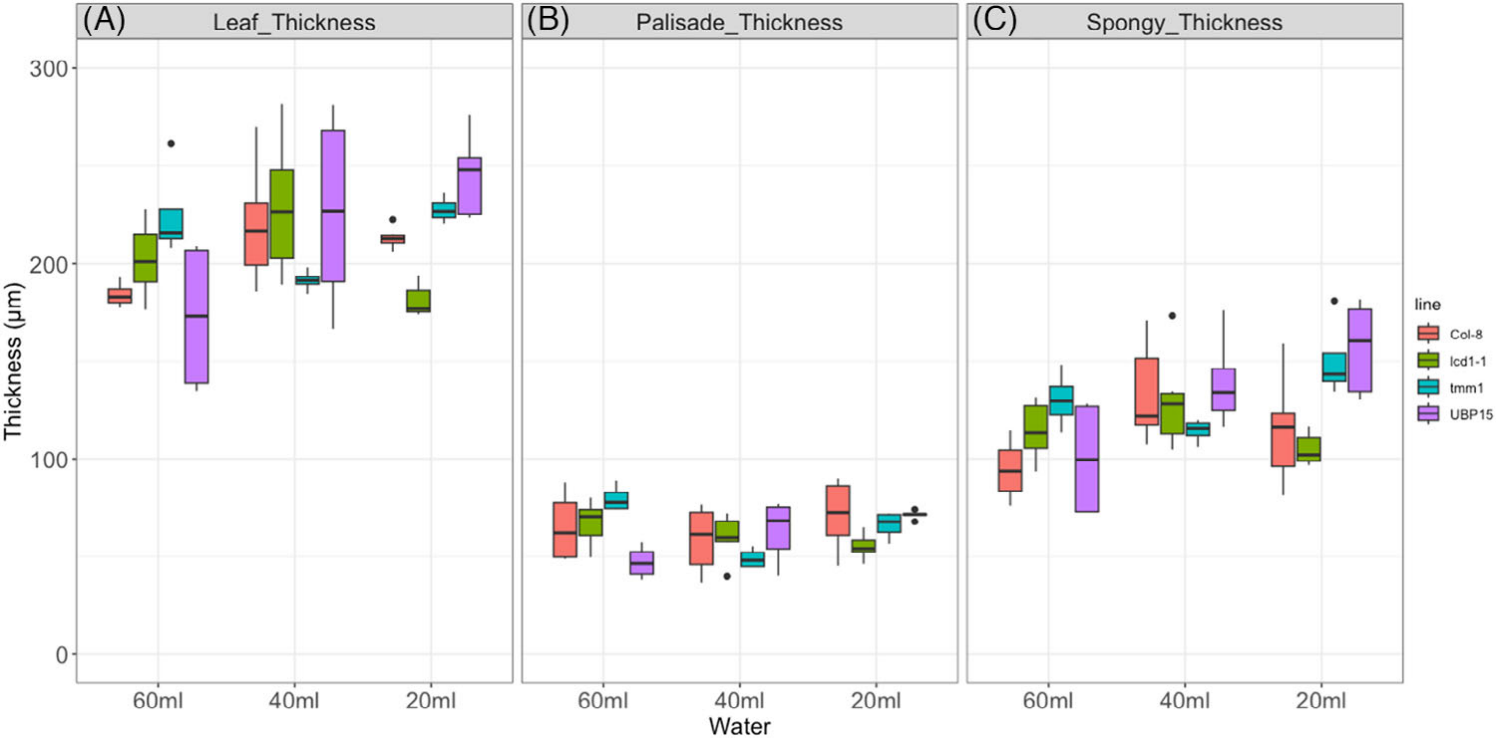
Comparison of A.t. genotypes after 7 days of reduced watering (‘short‐term stress’) showing thickness of (A) whole leaf, (B) of palisade parenchyma layer and (C) of spongy parenchyma layer. Water dosage was 60 mL, 40 mL and 20 mL, respectively. *n* = 12 measurements.

A comparison between the watering regimes of 60 mL (control), and reduced water dosage of 40 and 20 mL, respectively, after 7 days, is given in Figure 4. Already at the start of the experiments in fully watered plants, we noticed a striking difference in leaf thickness between the different genotypes. Col‐8 and UBP15 had the thinnest leaves while *tmm1* had the thickest leaves (Figure 4A, left plots). In the lines UBP15 and *tmm1*, this difference resulted mainly from the palisade layer, which is the thinnest and the thickest, respectively (Figure 4B, left plots).

The changes induced by reducing water from 60 to 40 mL in the thickness of the leaf in general, varied between the genotypes with little significance (Figure 4A). However, comparing 60 to 20 mL water dosage, we observed an overall increase in thickness in all lines apart from *lcd1‐1* (Figure 4A). In particular, UBP15 and *tmm1* had the thickest leaves when only given 20 mL of water. While in *lcd1‐1*, the reduced overall leaf thickness is due to the thinnest palisade layer, the great increase in thickness in line UBP15 resulted from the thick spongy parenchyma layer (Figure 4B and C).

Taking a closer look at each genotype, we see a significant increase in the leaf thickness from 60 to 40 mL and to 20 mL (Figure 4A). In the wild‐type genotype (Col8), the increased leaf thickness in moderately watered and severely stressed plants resulted from an increase in spongy parenchyma (Figure 4B) as there is no significant difference in the palisade layer (Figure 4B). The leaf thickness in *lcd1‐1* increased slightly from 60 to 40 mL watering but dropped significantly in severely stressed plants (20 mL water; Figure 4A right plots). The decreased leaf thickness of *lcd1‐1* rooted from a reduction in thickness of spongy parenchyma in the least watered plants (Figure 4C). Opposite to Col‐8 and *lcd1‐1*, the overall leaf thickness, palisade and spongy layers of *tmm1* decreased from 60 to 40 mL irrigation but interestingly, increased significantly in the plants that only got 20 mL of water (Figure 4A–C). The leaf thickness of UBP15 increased slightly while watering was reduced so that in severely stressed plants, it is significantly higher than in fully watered plants (Figure 4A–C). In UBP15, both palisade and spongy parenchyma layers increased significantly in severely stressed plants and caused the overall increase in thickness (Figure 4C).

### 3.3 Long‐term stress (12 days)

**FIGURE 5:**
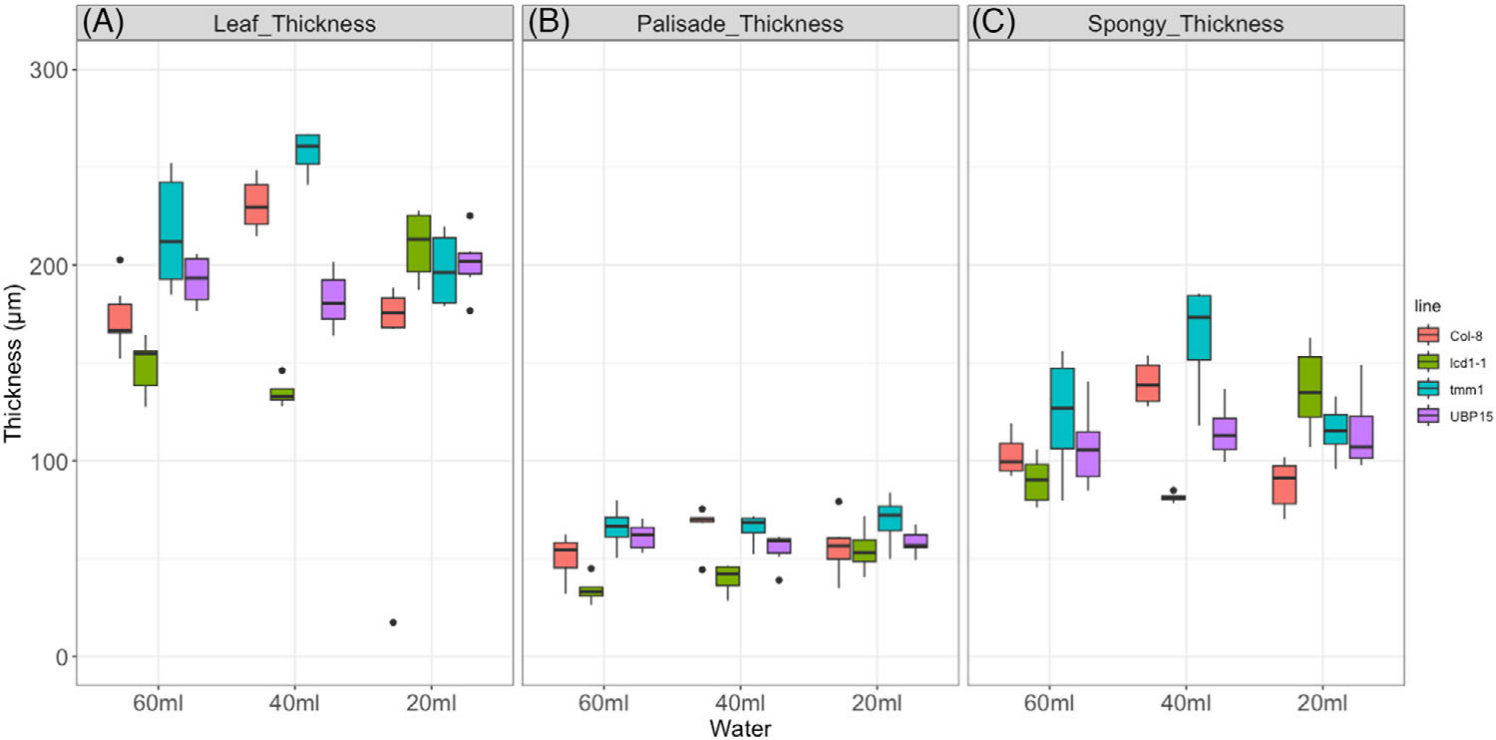
Comparison of A.t. genotypes after 12 days of reduced watering (‘long‐term stress’) showing thickness of (A) whole leaf, (B) of palisade parenchyma layer and (C) of spongy parenchyma layer. Water dosage was 60 mL, 40 mL and 20 mL, respectively. *n* = 12 measurements.

After 12 days of irrigation with 60 mL water, the overall thickness of the leaves clustered into two groups: *tmm1* and UBP15 had thicker leaves than the genotypes *lcd1‐1* and Col8 (Figure 5A, left plots). *tmm1* showed the highest leaf thickness as well as the thickest palisade and spongy parenchyma layers. Line *lcd1‐1* had the thinnest leaves overall and also the thinnest palisade layers in all treatments. However, in severely water‐stressed leaves (only 20 mL water), the spongy layer of *lcd1‐1* line significantly increased in thickness (Figure 5A–C). These two lines also differed most in the thickness of the spongy parenchyma with *tmm1* having the highest and *lcd1‐1* the lowest thickness of the spongy parenchyma layer (Figure 5C). Interestingly, under severe water stress, the high overall thickness of the leaf in *lcd1‐1* resulted from the spongy layer (Figure 5A and C) while in *tmm1*, the high overall thickness of the leaf resulted from the palisade layer (Figure 5A and B).

Similar to the short‐term stress, we see a significant increase in the leaf thickness from 60 to 40 mL and to 20 mL in long‐term stress (Figure 5A). The overall leaf thickness of Col‐8 increased significantly from 60 to 40 mL watering resulting from both, a significantly thicker spongy and palisade layer (Figure 5A–C). In severely stressed plants that only received 20 mL of water, the thickness of Col8 leaves decreased manly within the spongy parenchyma (Figure 5C), resulting in an overall thickness that is similar to 60 mL watering (Figure 5A, right plots). By contrast, in *lcd1‐1*, significant changes only occurred after switching from moderate (40 mL) to severe (20 mL) water stress, resulting in a significant increase in thickness in this line within the spongy parenchyma (Figure 5A and C). Also, in line *tmm1*, the palisade layer did not change by increasing water stress, but the overall leaf thickness increased in 40 mL water dosage as a result of increased thickness of the spongy parenchyma (Figure 5). In *UBP15*, the thickness of parenchyma layers remained almost constant by reducing the water dosage in long‐term water stress (Figure 5A–C).

### 3.4 Discussion

Drought is a severe challenge for plants, greatly reducing growth and yield. Here we looked at changes at the tissue level of four genotypes of *Arabidopsis thaliana* (*Col‐8*, *lcd1*, *tmm1* and UBP15) to evaluate the role of reduced water as well as the duration of this stress. To analyse the minute changes at the tissue level in drying leaves, high‐ resolution microscopy without re‐wetting the samples – as in conventional light microscopy – was essential. In conventional light microscopy at the tissue level, living material is usually prepared on slides in a water film or the samples are fixed and embedded in resin. However, for the present research question, any preparation in water or liquids was impossible as drought‐stressed cells could quickly regain full turgor in any liquid or fixative thereby distorting potential changes of cells by water loss and tissue shrinkage. To circumvent this dilemma, all leaves were plunge‐frozen and transferred directly to the SEM for imaging without any further treatment or contact to liquids.

Here, the investigated Arabidopsis lines reacted very differently and in particular, the layers of the mesophyll showed distinctive swelling and shrinking patterns. In general, most microscopic differences observed in the overall leaf thickness originated from the spongy parenchyma. Moderate water stress for a short time increased the leaf thickness in all genotypes except in *tmm1*, which can be related to the lack of trichomes in this genotype. Trichomes play a major role in mitigating the consequences of stress by conserving water in plants,^6^ adjusting the leaf temperature^31^ and/or protecting against light.^32^ An increase in leaf thickness in drought‐stressed plants has been observed before.^25^, ^33^, ^34^ However, the leaf thickness and specifically palisade thickness of *lcd1‐1* decreased significantly under severe stress. Due to the mutation, *lcd1‐1* produces less cells per leaf area.^35^ Therefore, it is speculated that *lcd1‐1* could compensate short‐term drought stress but was more susceptible to severe stress. The contrasting genotype to *lcd1‐1*, UBP15 with higher cells per leaf area, showed a significantly higher tolerance with almost no changes in leaf thickness over the course of the experiment.

Interestingly, *tmm1* compensated severe drought stress for the short‐term treatment very well but was susceptible when the stress persisted. On the other hand, the leaf thickness in this line increased in the medium water dosage and the longer stress period. Opposite to *tmm1*, leaves of *lcd1‐1* became thicker under severe stress in long‐term while in short‐time severe stress, the leaf thickness was less than in fully watered plants. In both *tmm1* and *lcd1‐1*, a significant variation in the spongy mesophyll layer was mainly responsible for the observed change in overall leaf thickness. This observation differentiates the thickness of palisade and spongy layers over time. It implies that in short‐term drought stress, the palisade layer is more affected by the water stress while in persisting, longer times of stress, the main variation occurs in the spongy layer. Thus, our results showed that the mesophyll plays a major role in drought tolerance of plant leaves, in particular the spongy mesophyll layer in long‐ and short‐term stress. Our data showed that the significant alteration (either increase or decrease) in leaf thickness resulted from changes in both spongy and palisade parenchyma. It is speculated that the elevated thickness of the spongy layer accelerates the rate of the photosynthesis by increasing the mesophyll conductance (*g*_m_).^36^ Therefore, developing thicker leaves by increasing the thickness of spongy and palisade layers is considered as an adaptation to drought stress to elevate the water use efficiency in low water availability conditions.^37^, ^38^

Due to their respective and specific leaf anatomy, the chosen genotypes reacted differently, for example, by a delay of this effect. Under severe stress conditions, the genotype UBP15 handled water stress better in short‐term stress while *lcd1‐1* showed increased mesophyll tissue thickness in long‐term stress condition. The *tmm1* genotype could at first increase the water content in this layer when being exposed to drought but shrinkage still occurred during the treatment period. The thick palisade layer in normal, bifacial leaf anatomy, as shown in control plants (Col‐8), also supported water loss in the beginning and the middle of the experiment; shrinkage as response to water shortage only occurred at the end of the severely stressed plants (20 mL water dosage).

The chosen genotypes with the respective anatomical differences help to evaluate our results of significant differences in mesophyll properties when plants are exposed to various levels of drought stress. We continue to analyse and relate these results to physiological data. We are aware of the fact that drought stress fuels the phytohormone synthetisation of abscisic acid (ABA), which regulates stomatal closure to avoid water loss.^39^ Furthermore, stressed plants produce elevated levels of reactive oxygen species (ROS) in their cells^40^ and the carbon metabolism in photosynthesis is also affected by a shortage in water supply.^41^ So, further studies regarding ABA, ROS or soluble sugar determination are necessary to link anatomical features and physiological responses triggered by drought conditions.

Here we described a novel technique with a focus on challenging sample preparation to image drought‐stressed leaves without manipulating the cell turgor pressure, which is inevitable in most light microscopy techniques. Our approach is an affordable and efficient method to visualise temporal and spatial alteration in mesophyll. We call for more studies applying this technique on other plants’ structures such as stems and roots.

## CONFLICT OF INTEREST STATEMENT

The authors declare no conflict of interest.
